# Anti-X Apron Wearing and Musculoskeletal Problems Among Healthcare Workers: A Systematic Scoping Review

**DOI:** 10.3390/ijerph17165877

**Published:** 2020-08-13

**Authors:** Maria Grazia Lourdes Monaco, Angela Carta, Tishad Tamhid, Stefano Porru

**Affiliations:** 1 Occupational Medicine Unit, University Hospital of Verona, 37134 Verona, Italy; angela.carta@univr.it (A.C.); stefano.porru@univr.it (S.P.); 2 Department of Diagnostics and Public Health, Section of Occupational Health, University of Verona, 37134 Verona, Italy; 3 Postgraduate School of Occupational Medicine, University of Verona, 37134 Verona, Italy; tishad.tamhid@studenti.univr.it

**Keywords:** anti-X apron, lead apron, musculoskeletal disorders, interventional radiology, radiation protection

## Abstract

Interventional radiology activities and other medical practices using ionising radiation have become increasingly prevalent. In this context, the use of anti-X aprons, in association with awkward postures and non-ergonomic working conditions, might cause the onset of musculoskeletal disorders (MSDs). This research aims to evaluate the evidence about the correlation between wearing anti-X aprons and work-related MSDs. A systematic scoping review of articles published between 1990 and 2020 was conducted by searching the PubMed, Scopus, Embase, and Web of Science databases. Twelve cross-sectional studies, conducted among interventional physicians, nurses, and technicians, were finally included. Five studies primarily investigated the association between use of anti-X aprons and MSDs, showing that a higher prevalence of disorders was not always associated with the use of protective aprons. No studies investigated the impact of anti-X aprons on fitness for work assessment, particularly in subjects with MSDs. There is no complete agreement about the correlation between anti-X apron-wearing and the occurrence of MSDs, although the possible discomfort of workers using anti-X aprons appears more evident. Further studies are needed to objectify the role of these protective devices in the genesis of MSDs and to offer specific ergonomic solutions for healthcare workers.

## 1. Introduction

The widespread use of X-radiation has led, over the years, to a need to investigate aspects related to the safety and protection of healthcare workers (HCWs). The risks involved in its use, especially in interventional procedures, favoured the evolution and diversification of relative individual protection devices. The anti-X apron is one of the most important personal protection devices for HCWs who are potentially exposed to radiation, along with neck shields, gloves, and lead-impregnated glasses [1]. 

Historically, radiation-shielding materials have been made of lead due to its great attenuating qualities. The radiation protection of garments is indicated by lead equivalence, with 0.5 mm lead aprons considered the standard, which attenuates more than 95% of incident radiation [1,2]. A lighter, thinner, lead-equivalent garment made of materials differing from lead may provide adequate protection, as a 0.3 mm lead-equivalent apron will result in only a modest increase in the effective dose (7–15%) compared with a 0.5 mm lead-equivalent garment. Currently, other materials such as barium, bismuth, and antimony are used, which are available in mixtures with low lead content or as lead-free. More significant lead equivalence increases protection but does so at the cost of increased weight [2]. Recent innovations have aimed at methods for decreasing the weight burden, considering the related skeleton injuries associated with load-bearing procedures. Several manufacturers have come out with reduced weight, non-lead radiation protection aprons. Therefore, an essential aspect is to guarantee an appropriate balance between comfort and protection in order to reduce the strain without compromising safety [1]; however, this aspect is not always achievable. Generally, lead aprons do not attain the dual goals of excellent radiation protection and musculoskeletal preservation and, when made lighter to help aching spines, fall below acceptable protection standards [2]. Lightweight aprons may be satisfactory for some ancillary personnel who frequently stay behind a barrier or wall, but not for first operators [3].

There are various categories of anti-X aprons: closed (shielding material all around) vs. open backs (absence of shielding materials behind), one-piece vs. two-piece (e.g., apron vs. skirt and vest), and belted vs. unbelted one-piece [3]. The old-fashioned, unbelted, one-piece overcoat style with a closed back causes more overload to the upper body, and the HCWs who wear it often refer to have troubles in this area [3]. To date, there do not yet exist clear guidelines about this specific issue. 

The International Standard IEC 61331 (International Electrotechnical Commission) Part 3 applies to protective devices against diagnostic medical X-radiation, which deals with general requirements in terms of design, materials used, and minimum attenuation properties of materials, but lacks further ergonomic indications [4].

Anti-X protective clothing is effective in reducing the dose of exposure to X-rays, but could have the disadvantage of still being overloading, with a possible negative effect on the musculoskeletal system, primarily with respect to the spine [1,3,5,6]. 

To date, there is no unequivocal etiological hypothesis about the relationship between wearing lead aprons and onset of musculoskeletal disorders (MSDs). A first hypothesis is related to the whole-body biomechanical overload caused by aprons, which can weigh from about 3–4 kg up to 7–8 kg, depending on their size. The overload could mainly cause the discomfort and fatigue perceived by workers. Moreover, wearing aprons has been associated with decreased ease of movement, which could limit joint excursions and affect the possibility of changing posture. Furthermore, concerning posture, interventional procedures may involve prolonged and fixed standing postures lasting eight or more hours. The coexistence of all these factors could explain the whole-body or specific side pain (e.g., neck pain or back pain) referred to by the workers [3,5].

As a consequence, pain, discomfort, physical strain, or other musculoskeletal disorders (MSDs) could result in increases in absenteeism, need for antalgic treatments and, in some cases, in job restriction in HCWs, particularly for workers already affected by musculoskeletal problems (e.g., disc hernias, rheumatic diseases, osteoporosis, and so on). Nonetheless, the real correlation between wearing a lead vest/apron and MSDs remains controversial. 

This systematic scoping review aims to provide a basis for targeted research regarding the relationship between the use of anti-X aprons and musculoskeletal problems among HCWs.

## 2. Materials and Methods 

Preferred Reporting Items for Systematic Reviews and Meta-Analyses- Scoping Reviews (PRISMA-ScR) guidelines on conducting systematic scoping reviews were followed [7]. 

### 2.1. Research Question

The research question for this review was: ‘What is known from the current literature about the use of anti-X aprons and the frequency of MSDs among healthcare workers?’.

The results of this research were summarised and discussed to answer the following questions:

(1) Is there an association between the use of anti-X aprons and the onset of musculoskeletal disorders?
(2) Are there specific factors underlying this association?

### 2.2. Eligibility 

References were screened by setting the database parameters to English language, healthcare setting, and study type (original articles published in the peer-reviewed journal). 

### 2.3. Literature Search Strategy 

Studies published between January 1990 and March 2020 were searched for in PubMed, Scopus, Web of Science, and Embase. The following keywords were used: ‘lead aprons’ OR ‘lead apron’ OR ‘radiological shielding garment’ OR ‘radiological shielding garments’ OR ‘personal radiation protective apparel’ OR ‘personal radiation protective apparels’ OR ‘radiation protective apron’ OR ‘radiation protective aprons’ OR ‘radiation protection shield’ OR ‘radiation protection shields’.

Additional data sources included the authors’ pre-existing knowledge of the literature and manual review of reference lists of studies. 

### 2.4. Study Selection Process

Once both members (M.G.L.M. and T.T.) read all abstracts and selected relevant articles, a comparison between those selected occurred. If there was a discrepancy in an article selection, a discussion to reach consensus ensued with a third author (A.C.). The researchers revisited the inclusion and exclusion criteria and used these as guidelines for determining whether the articles should be included.

### 2.5. Data Extraction

A data extraction form was developed to determine which variables to extract. The following items were included:

- Article identifiers (authors, year of publication)
- Study identifiers (sample size, design, country)
- Aim of the study
- Features of the participants (e.g., sex, age, job title)
- Main results
- Anti-X aprons characteristics
- Anti-X aprons usage time
- Musculoskeletal disorders or diseases
- Job fitness

### 2.6. Summarising and Reporting the Results

A qualitative description of the included studies can be found below. Tables and diagrams are used to synthesise the main findings. 

To evaluate the methodological quality of the studies included, the Critical Appraisal Skills Program (CASP) checklist for the reporting of all qualitative studies has been applied [8]. The coder will be asked to record a “Yes”, “No”, or “Can’t tell” response. According to this approach, two authors (M.G.L.M. and T.T.) independently fulfilled a ten-item questionnaire in order to evaluate, for each article, these issues: study validity (aim, study design, sampling), data analysis, and clinical relevance and generalisability of study (data interpretation and findings disclosure). Disagreements, like the procedure above, will be resolved via discussion between two authors, and, if required, a third reviewer’s (A.C.) opinion will be sought. Quality analysis was summarised, in a descriptive way, in the Results Section. 

## 3. Results

### 3.1. Selection of Sources of Evidence, Study Characteristics, and Settings

**Figure 1:**
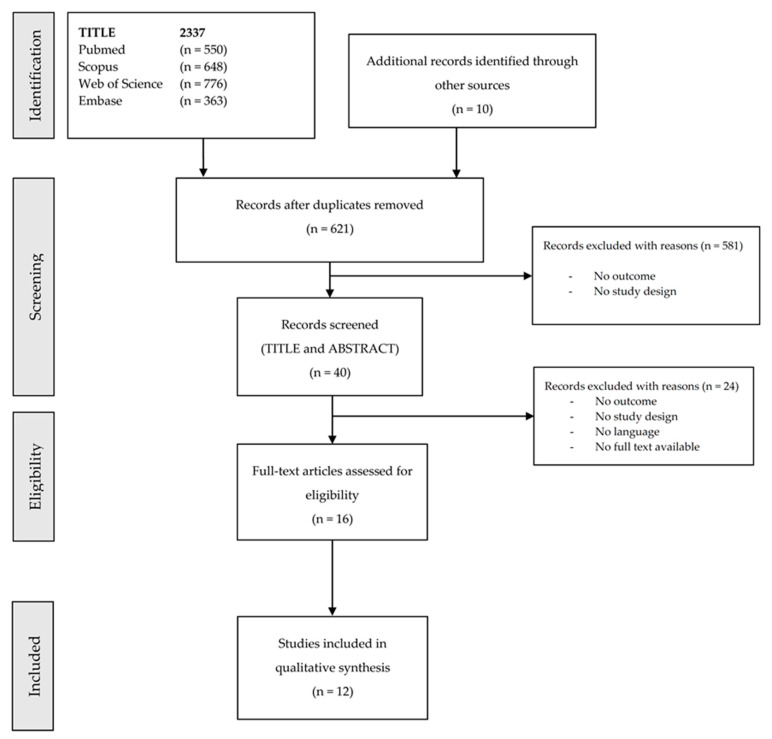
Flow diagram depicting the different phases of the systematic scoping review following the Preferred Reporting Items for Systematic Reviews and Meta-Analyses (PRISMA) statement.

As shown in the flow diagram reported in Figure 1, a total of 2337 articles were retrieved. Of these, twelve studies [9,10,11,12,13,14,15,16,17,18,19,20] were finally included. All studies had a cross-sectional design. Three studies used instrumental measurements, conducting laboratory experiments on healthy volunteers [13,20] or in a real working setting [9], while the other studies were based on anamnestic surveys. 

### 3.2. Study Outcomes 

Only five of the studies included had, as a main objective, the possible associations between use of the anti-X apron and MSDs. Overall, they showed a higher prevalence of MSDs among interventional physicians enrolled, which was not always associated with the use of protective aprons. Only five studies also evaluated the type of gown (whole or two pieces) or its weight. No studies directly evaluated the rates of MSDs by apron design. No studies concerning the use of anti-X aprons by workers already affected by MSDs were found. None of the studies investigated the impact of anti-X gowns in assessing fitness for work, particularly in subjects with MSDs.

The methods and relevant findings of published papers included in this review are summarised in Table 1.

### 3.3. Study Quality

The studies included in this review showed an overall medium quality level, with regard to the main characteristics related to their purpose, methodology, result reporting, and generalisability. On one hand, almost all studies clarified the purpose of their research; on the other hand, the sample size varied remarkably (from four to more than a thousand participants) and was not comparable between the several studies, as well as the data collection methods. Only three papers [9,13,20] included “measurements” of physiological or anthropometric parameters, while almost all of the studies consisted of data collection, mostly self-administered and with non-validated questionnaires. These characteristics constitute a critical quality limit.

Another aspect to consider in the overall quality assessment is the presence of a control group: only four studies used a control group [9,10,17,18] and, in one case, the subjects performed their activities with and without wearing aprons.

A further issue lowering the quality level of the included studies is related to endpoints. Even if the outcome was a disease (e.g., disc herniation), in several cases, it was assessed only through medical history and not by clinical or objective instrumental examination.

Finally, as for data reporting, all the articles showed a reasonable level of quality. 

## 4. Discussion

Interventional procedures using ionising radiations are now widespread in the medical field. They involve several specialists, such as radiologists, cardiologists, vascular surgeons, gastroenterologists, and urologists, that expose themselves to non-negligible doses of X-rays and, therefore, require specific collective and individual protective devices. Furthermore, long-lasting procedures obligate physicians to assume an awkward and prolonged posture, not only of the spine (e.g., an erect posture or cervical spine flexion), but also of the upper limbs. All these aspects should be considered to evaluate the ergonomics of these working tasks and to improve them. A specific trait is that the complexity of these tasks makes it difficult to distinguish the contribution of each component in the genesis of biomechanical overload and MSD onset in the HCWs exposed.

Nevertheless, almost all the workers who participate in a procedure that involves the overuse of fluoroscopy undoubtedly recognise the use of anti-X aprons in healthcare settings as a factor of physical stress. ‘Lead’, a name often used to indicate the anti-X aprons, is considered a leading cause of discomfort, fatigue, and myalgias.

This review aimed to systematically summarise the state-of-the-art on the link between the use of anti-X aprons and MSDs among HCWs. Despite being a topic of daily interest for the occupational physician who deals with radiation protection, the literature related to this issue is rather scarce, and the studies that do exist in the literature have provided heterogeneous results. 

Based on the results obtained, the answers to the research questions of this review are shown below.

### 4.1. Research Question #1: Is there an Association between the Use of Anti-X Aprons and the Onset of Musculoskeletal Disorders?

Many studies included in this review suggested that overuse of anti-X aprons is associated with possible occupational health risks, especially with a high prevalence of musculoskeletal problems (particularly those related to the spine). They also suggested that the use of aprons is not the only cause: The combination of axial load (i.e., prolonged standing or sitting in non-ergonomic positions while carrying the load), awkward or poor posture (as necessitated by leaning or bending to accomplish procedures), and repetitive injury accumulated over years of practice could participate in the onset of MSDs [2,6]. These contributing factors for back and neck injuries may be aggravated by age-related changes in the muscles, vertebrae, and intervertebral discs, as well as by common pre-existing causes of mechanical neck and back pain. 

#### 4.1.1. Spine Diseases

To date, there have been very few published studies looking specifically at the development of MSDs in interventional physicians related to the use of anti-X aprons. Most of the included studies assessed that the rates of neck and back disease were increased in interventional physicians and angiographers.

Ross et al. [18] found an elevated rate of cervical disc herniation and multiple level disc diseases occurring in physicians using lead aprons. These notable findings prompted the development of the term ‘interventional disc disease’.

Goldstein et al. [12], in 2004, gathered support for specific patterns of disease and injury within interventional proceduralists. In their survey, there was a high prevalence of a history of musculoskeletal problems (42% of responders complained of spinal disorders, with 70% described as lumbosacral and 40% as cervical). In this same study, the number of years performing invasive procedures post-fellowship was found to be significantly related to having spine disorders: 26% of physicians with less than five years of experience reported spinal problems, while this percentage increased to 60% in those physicians who had more than 21 years of interventional practice. No specific relationship between the rate of orthopaedic injuries and yearly caseload was identified [14].

A survey conducted in 2011 among interventional electrophysiologists in Canada by Birnie et al. [10] showed a higher prevalence of lumbar spondylosis, although not statistically significant. Nevertheless, there surprisingly emerged a much higher prevalence of cervical spondylosis (statistically significant) for HCWs who performed interventional procedures vs. those who did not.

Moreover, in the study conducted in 2011 by Elkoushy et al. [11] among endourologists, all musculoskeletal complaints (multivariate) correlated with the annual combined caseload of ureteroscopy and percutaneous nephrolithotomy.

The survey among the members of The Society of Cardiovascular Angiography and Interventions (SCAI) conducted by Klein et al. confirmed the conclusion of the previous 2004 SCAI survey, reporting a relatively high prevalence of spine injuries among interventional cardiologists and staff using anti-X aprons [21]. 

#### 4.1.2. Musculoskeletal Pain

As regards the high frequency of back pain (BP) and neck pain (NP) among interventional physicians using X-rays (radiologists, cardiologists, electrophysiologists, and endoscopists), it cannot immediately be determined whether the use of anti-X aprons defines the pain in the operators or not [2]. 

The study conducted in 1991 among radiologists by Moore et al. [15] did not show a strong statistical correlation between anti-X apron use and higher complaints of low back pain (LBP), although more than half of frequent lead apron users complained of LBP and those who wore aprons for more than 10 h a week missed more time from work because of the pain. 

In 1997, Ross et al. [18] compared work-related injuries and trends of disease among different specialties, such as rheumatology (standing for short periods while examining patients), orthopedic surgery (standing for long periods at an operating table without weights), and interventional cardiologists (standing for long periods while wearing lead aprons). Interventional cardiologists use anti-X aprons in a majority of procedures, while orthopedists spend a smaller fraction of procedures wearing leaded garments. After stratifying by the use of the lead aprons, interventional cardiologists reported significantly more musculoskeletal problems then orthopaedics and rheumatologists. Increased NP and BP in interventional cardiologists were not directly correlated to procedural or operative times. 

In the study conducted in 2002 by O’Sullivan et al. [15] among Canadian Endoscopic retrograde Cholangiopancreatography (ERCP) endoscopists, almost all of them attributed BP and NP symptoms to ERCP (the majority wearing a one-piece lead apron while performing them). 

The key results of a large study at the Mayo medical centre in 2015 (Orme et al.) [17] demonstrated that work-related musculoskeletal pain was significantly more common among HCWs participating in interventional procedures, compared with those who did not. This study showed a significant association between a history of work-related pain and occupational exposure to fluoroscopically guided procedures requiring lead aprons, with a 67% increase in the prevalence of musculoskeletal pain. 

In the most recent study, Livingstone et al. [14] provided radiology interventionists with a questionnaire that included age, area of expertise, years of experience, weight and type of anti-X apron used, and the problems caused by the use of aprons. Almost half reported that they had body aches due to wearing single-sided aprons. Interventionists who performed more than ten hours per day wearing a one-piece anti-X apron, as predicted, principally complained of shoulder pain and back pain. 

Therefore, while the high prevalence of musculoskeletal pain among HCWs involved in interventional radiological activities appears evident, it is not evident that this disorder could be due to the use of anti-X aprons.

#### 4.1.3. Discomfort and Ease of Movement

Although there is no complete agreement about the correlation between anti-X apron-wearing and the occurrence of MSDs, possible worker discomfort when using the anti-X apron is clear. All included studies highlighted discomfort, fatigue, or awkwardness of movement of some sort among HCWs using the anti-X apron. 

In particular, the pilot study conducted by Rothmore [19] in 2002 compared body part discomfort ratings, fatigue, and ease of movement among five radiographers while wearing two-piece lead suits, one-piece suits, and one-piece suits with waist belts. In this study, subjects were asked to complete ‘Visual analogue scales’ for discomfort immediately before wearing their protective anti-X aprons and again after the procedural list. Despite the limitations of the study size, significant discomfort increases in the neck, shoulders, and lumbar spine, as well as general fatigue, were found among subjects while wearing a one-piece anti-X apron. Even though not reaching significant levels, trends towards increasing thoracic discomfort and decreased ease of movement were also noted. No significant differences were found between two-piece suits and one-piece suits with waist belts. This pilot study once again highlighted the importance of the correct choice of lead apron among those who use them as an occupational tool.

Finally, despite the relevance of the topic, no studies concerning the use of anti-X aprons by HCWs already affected by MSDs were found. However, the type, weight, and time of use of this equipment could be assessed for the fitness of the workers exposed to ionising radiation. 

#### 4.1.4. Muscular Activity and Fatigue

Only three studies were conducted using an instrumental method to investigate muscular activity and fatigue during anti-X apron wearing.

The purpose of the study conducted by Alexandre et al. was to quantify the impact of wearing anti-X aprons on the discomfort and fatigue of surgeons practising gastrointestinal endoscopies, using infrared thermography. They compared two situations: (a) the classic endoscopy department (without apron) and (b) the operating room with the apron. Their results demonstrated that the trunk muscles, when covered by an anti-X apron, were significantly recruited during all procedures, with consequent discomfort and fatigue for HCWs [9].

Johnson et al. [13] tested the hypothesis that wearing the 3.7 kg vest portion of a radiological shielding garment significantly increases lower back and shoulder muscle activity in quasi-static erect and forward-flexed postures. The outcome was investigated using surface electromyography (sEMG). This study showed that the use of a lead vest for a short time does not significantly increase muscle activity in the lower back and shoulders. Nevertheless, the study had too many limitations to be conclusive, such as the short term of measurements and the type of participants (only healthy subjects).

Recently, Tetteh et al. [20] published an experimental study on sixteen healthy voluntary participants using sEMG during the simulation of a catheterisation procedure under fluoroscopic guidance, in order to examine the effects of protective aprons on fatigue onset of the erector spinae and trapezius muscles. According to the results of this study, wearing protective radiation aprons significantly accelerated fatigue development in several muscle samples.

### 4.2. Research Question #2: Are there Specific Factors Underlying these Associations?

The studies reported different characteristics of the workers (e.g., age and gender) and work activity (e.g., profession, workload, and type of gowns used). However, the association between these factors and the development of MSDs in workers who used anti-X aprons was not investigated in all of these cases.

As for gender, it could not be associated with the frequency of musculoskeletal disorders. This aspect, however, was not sufficiently investigated in all the studies, in which (among other things) a high predominance of males emerged, reflecting the more frequent male presence in interventional activities. In the few studies presenting a gender-balanced sample, the sample size was too small [9,19] or consisted of healthy volunteers [13]. Only one study [17] reported that female gender is associated with a higher prevalence of musculoskeletal pain. However, data may have been affected by the fact that these were mainly technicians and nurses, in whom it is not possible to exclude overload also deriving from other factors (e.g., manual patient handling). Overall, in this study, males represented only a third of the sample (selection bias).

As for age, not all studies considered this factor in their statistical analyses. Overall, there were no statistically significant differences in the jobs that took this aspect into consideration [10,17], except in some cases where age was associated with greater seniority [11,12] and, as a consequence, with hours or years working with anti-X aprons.

The type of apron (one-piece vs. two pieces), as well as its weight and wearability, could influence the likelihood of muscular impairment and pain, which must be taken into account in an ergonomic evaluation. However, this aspect was poorly investigated in the selected studies. Ross et al. [18] found that the higher rate of discomfort reported by cardiologists may be due to greater pressures generated within the discs while supporting the weight of the one-piece suits directly through the shoulder girdle. 

According to this research, Benjamin et al. [5] did not find any studies that directly evaluated the rates of MSDs design. The authors suggest that almost one-half of users said their protective garments were uncomfortable: two-piece garments—the skirt and vest configuration—may reduce loading pressure on the cervical and thoracic spine. 

Not all these considerations, however, can be made without considering the worker’s anthropometric characteristics, as well as their physiological and pathological conditions. If the use of a two-piece apron can reduce cervical spine and neck muscles’ overloads, it can be perceived that it could represent an insult in a subject affected by lumbar spine or lower limb pathologies (e.g., coxarthrosis). Moreover, when the skirt is used, it rests on the pelvic girdle and the iliac crests, causing discomfort. Furthermore, the weight of a two-piece apron can be overall higher than that of a single-piece apron.

Considering the weight of the lead aprons, it varied according to the size, thickness, and material from which they are made. A large study conducted by Buchanan et al. [22] in 2012, enquiring among interventional personnel, showed that only 19.5% had musculoskeletal problems (back/neck/hip pain). However, an association between these problems and years of exposure was found. Such a finding could be associated with improvements in the ergonomic design of radiation protection apparel, which have become more lightweight and which may distribute the weight of the protection more evenly.

As a result of the heterogeneity of the research works, the findings of this review should be interpreted with caution.

### 4.3. Aetiological Hypotheses and Proposal for Further Research

As high-complexity procedures continue to develop, an increasing number of HCWs must wear an anti-X apron for an extensive period of time, in situations which are often associated with awkward postures. The evidence emerging from this review, despite the qualitative limits of the included studies, offers interesting insights to explain the correlation between wearing aprons and MSDs.

A possible pathophysiological mechanism could be the prolonged compression of the intervertebral discs and the increased intramuscular pressure, which can cause a local reduction in blood flow and an inadequate supply of nutrients and, in the same way, a lack of reduction of waste products [23,24]. In the long run, this might act in parallel with environmental or genetic factors to accelerate degenerative processes. Such modifications could be more significant in senior workers or subjects already affected by musculoskeletal diseases.

Starting from these assumptions and considering the weaknesses of the current literature, laboratory and field studies are needed to isolate, as far as possible, the sole effect of anti-X aprons on whole-body biomechanical overloading, distinguishing it from other factors (e.g., posture, microclimatic conditions, comorbidity of workers, and so on). 

To this end, the combined use of several instrumental methods, which have already been individually tested by some reserchers, could provide specific information [9,13,20].

Methodologically, it is essential to carry out prospective longitudinal studies with adequate sample size and, if possible, with control groups (according to ethics), in order to provide generalisable and useful results in real working conditions. These studies could help to understand the contributions of modifiable and non-modifiable factors (such as age and gender) in the pathogenesis of these disorders.

Rehabilitation interventions, both including physical activity promotion and return-to-work programs, could be an interesting further line of research. In fact, it should be kept in mind that muscular tone and joint function are indispensable in balancing occupational and leisure-time activity biomechanical overload. 

Last, but not least, no studies investigated the risk perception and knowledge of workers about occupational health and safety issues. These aspects need to be explored in depth, in order to understand how any incorrect and modifiable behaviours can contribute to the onset of MSDs.

### 4.4. Strengths and Weaknesses

The strength of this scoping review is the methodology through which the study was conducted. Four databases were consulted using a sensitive search string, in order to select the most significant number of articles related to the research topic. Two reviewers conducted the article selection double-blind, while a third reviewer resolved the disagreements. Only articles published in peer-reviewed journals were included. The research period is quite vast, covering the past 30 years.

A weakness can be attributed to the small number of selected studies and to the fact that these studies were conducted with different protocols and were mostly based on the collection of anamnestic data. 

The different and overall medium quality of the included studies represents another weakness of this review. 

All methodological limits found and described above are inevitably reflected in the quality of conclusions.

In several studies, the questions of the survey were reliant upon self-reporting of illness without any documentation or confirmation, and often the surveys’ results were closed (i.e., not allowing for multiple responses), forcing a reduced contextualisation of the answer. Also, it should be acknowledged that pain is a subjective symptom. Many HCWs referring to back pain could have no radiologic or other objective evidence of pathologic change. Individual variation in pain perception is unavoidable. 

Additionally, this search had a large time frame and it is likely that the anti-X aprons considered in earlier studies (which were heaver and thickly leaded) are not used anymore. 

## 5. Conclusions

The diffusion of interventional radiology practices, often long-lasting and forcing HCWs to maintain awkward postures, is associated with frequent and long periods of anti-X apron use. The scientific evidence regarding this topic does not provide uniquely interpretable results. Further laboratory and in-field research is necessary to objectify the role of these protective devices in the genesis of MSDs and to offer specific ergonomic solutions for HCWs. 

Several measures emerging from the data proposed in this study can be taken to prevent or lessen MSDs for HCWs. In this context of applied research and clinical practice, the role of the Occupational Physician is aimed at helping to protect the health and fitness of HCWs. The results of this scoping review will be used as a basis for more targeted studies in the future.

## Figures and Tables

**Table 1:** Main features and results of the 12 studies included in the scoping review about the correlation between anti-X apron use and musculoskeletal disorders (MSDs) onset. Study conclusions, relating to the topic of this review, are highlighted in bold.

Author, year [Ref.no] Country	Study Design/Aims/Objectives	Setting/Population/Sample	Methods	Main Results
**Alexandre et al., 2017** [9] France	Cross-sectional study To quantify the impact of the weight of radiation protection lead aprons on the discomfort and fatigue of HCWs.	In the field Four HCWs (2 F; 2 M) Interventional gastroenterology operating room with and without use of X-rays.	Muscular discomfort and fatigue Skin temperature measured by Infrared thermography Five muscle groups were investigated: deltoid, Pectoralis major, Trapezius, Lumbar spine, Hamstring muscles Three different situations: ▪ after the reference situation (T0), ▪ after 3 h spent in the classical endoscopy service (without apron) (T1), ▪ after 3 h spent in the operating room with apron (T2).	All the muscular groups studied, especially trapezoids and pectorals, had significant temperature increases, with discomfort and fatigue inducing back pain in medical staff. **The apron weight is carried primarily by the shoulders, symmetrically, and the corresponding muscle groups may have a contraction threshold that is best suited for resisting this weight, even in static position.**
**Birnie et al., 2011** [10] Canada	Cross-sectional study To determine the prevalence of cervical and lumbar spondylosis in a group of interventional electrophysiologists, in comparison to a control group of non-interventional cardiologists. To examine the potential predictors of the development of disease. To investigate current practices of ergonomic planning of electrophysiology laboratory and ergonomic training of electrophysiologists.	By web survey 58 Interventional electrophysiologists - Mean age 45.66 ± 9.63 y; - 94.8% Male. 36 Non-interventional Cardiologists - Mean age 46.31 ± 7.74 y; - 94.4% Male.	Web-based Survey conducted with an online questionnairre, consisting of three sections: - The first section asked for baseline demographics, years of clinical practice, and details of electrophysiology laboratory practice (including the number of hours per week of wearing lead and type of lead). - The second section asked about symptoms of cervical or lumbar spondyolosis. - The last section contained questions about morbidity from and disease treatment.	There was a significantly higher prevalence of cervical spondylosis in the electrophysiologists (20.7% compared to 5.5%, *p* = 0.033). There was a trend for increased prevalence of lumbarspondylosis (25.9% compared to 16.7%, *p* = 0.298). **No significant difference related to lead-wearing between HCWs affected or non-affected by cervical and lumbar spondylosis.**
**Elkoushy et al., 2011** [11] Canada	Cross-sectional study To assess the compliance of endourologists with radiation safety measures To determine the prevalence of orthopedic complaints.	By web survey 134 Endourologists Age - <40 y 33 (24.6%) - 40–60 y 85 (63.4%) - >60 y 16 (11.9%)	An Internet-based survey was sent to all members of the Endourological Society. Baseline characteristics of practice patterns, compliance with various radiation protection measures, and prevalence of various orthopedic compliants were assessed. Open-ended questions assessed specific orthopedic compliants and reasons for non-compliance with radiation safety measures.	64.2% (*n* = 86) reported muscoloskeletal problems: 38.1% (*n* = 51) back problems, 27.6% (*n* = 37) neck problems, and 17.2% (*n* = 23) hand problems. **26.5% of endourologists enrolled cited discomfort and heaviness of lead aprons as reason for non-compliance in wearing them.**
**Goldstein et al., 2004** [12] USA	Cross-sectional study To characterise the prevalence of orthopedic and radiation-related health problems among invasive cardiologists in contemporary practice.	By web survey 424 Interventional cardiologists	A Web-based Survey was sent to the Society for Cardiac Angiography and Interventions members. Health questions (yes/no) focused on orthopedic problems (spine, hips, knees, and ankles) and problems associated with chronic radiation exposure.	60% (*n* = 96) of physicians performing invasive procedures with more than 21 years experience reported spine problems (*p* < 0.05). Hip, knee, or ankle problems were noted in 28% of operators. **Authors related spine problems, at least in part, to a greater number of hours bearing lead.**
**Johnson et al., 2011** [13] USA	Cross-sectional study The primary goal of this study was to test the hypothesis that wearing the 3.7 kg vest portion of a radiological shielding garment (a ‘lead’) significantly increases lower back and shoulder muscle activity in quasistatic erect and forward-flexed postures. Secondarily, the authors examined the effects of gender and forward-flexed posture, as well as their interactions with lead use.	Laboratory 19 young healthy adults (9 Female; 10 Male) Age range 21–30 y	sEMG recording of muscle activity of trapezius and back muscle groups. For each muscle group, a two-group (by gender) repeated measures study with two within-subject factors (erect or forward-flexed posture, presence or absence of the vest) was performed. Filling out a questionnaire on which participants described their perceived level of effort and discomfort in postures with and without the lead using graphic rating scales.	**Use of the lead did not result in a significant increase in muscle activity in the lower back or shoulders, despite perceived increases in effort and discomfort.** Posture proved to be the most significant secondary factor affecting activity in the lower back, while participant gender proved insignificant. **Short-term use of the lead does not appear to contribute to the incidence of back pain or injury in interventionalists.** Avoiding flexed postures could more directly reduce the likelihood of pain or injury.
**Livingstone et al., 2018** [14] India	Cross-sectional study To evaluate the knowledge and practice of using radiation-protective aprons by interventionists in radiology.	Questionnaire 91 Vascular and Interventional Radiologists Age range - 30–40 y 44% - 40–50 y 29%	Paper-and-Pencil Self-Administered Simple Survey with demographic, occupational, and health questions about type and duration of interventions; model, material, type, and weight of apron used, and health problems	**47% prevalence of body aches attributed to wearing single-sided aprons.** **Most of the interventionists who wore lead-free aprons did not complain of any physical strain.**
**Moore et al., 1991** [15] USA	Cross-sectional study To investigate the possibility that wearing lead aprons during interventional radiology procedures might be a vocational risk factor for back pain.	By e-mail survey 236 Radiologists (Gastrointestinal, cardiovascular, and interventional radiologists) respondend - 25 F; 211 M - Age range 30–67 y - 179 subjects finally enrolled	Four-part, 23-item questionnaire - 1st part: general information - 2nd part: use of lead aprons (average number of hours per week, total number per years) - 3rd part: experience of back pain and if its onset predated the use of lead aprons - 4th part: alleged association between onset and persistence of back pain and lead apron use.	52% prevalence of back pain in those who reported to use lead aprons frequently, compared with 46% in those who use infrequently; (OR_M-H_ =1.18 [0.64–2.15]). Severe back pain was reported by 12% of frequent apron users and 8% of infrequent apron users (OR_M-H_ = 1.61 with 95% confidence limits of 0.55 and 4.68). Back pain was reported by 49% of long-term apron users and 48% of non-long-term apron users (OR_M-H_ = 0.83 with 95% confidence limits of 0.43 and 1.59). Severe back pain was reported by 12% of long-term apron users as opposed to 7% of non-long-term apron users (OR_M-H_ = 2.29 with 95% confidence limits of 0.66 and 7.92). Of those respondents who first experienced back pain after they began to wear a lead apron, 43% (33/76) thought that the apron was at least partly responsible for their symptoms. Of all respondents with back pain, 49% (62/127) reported that their pain worsened when they used a lead apron. Back pain caused 24% (32/131) of all respondents with back pain to consciously limit the amount of time spent wearing a lead apron and led 7% (9/128) to consider a change in subspecialty. **Authors concluded that, although many radiologists thought that lead aprons played a role in the development of their back pain, their study did not show a statistically significant association.**
**O’Sullivan et al., 2002** [16] Canada	Cross-sectional study To examine the practices of Endoscopic retrograde Cholangiopancreatography (ERCP) and the prevalence of musculoskeletal injuries.	By mail survey 114 endoscopist practising ERCP	Paper-and-Pencil Self-Administered Questionnaire on: - ERCP practices - -musculoskeletal conditions experienced and questions related to them - physical risk involved in performing ERCP, such as the type pf lead aprons worn, the type of endoscope used, and the frequency of breaks (defined by removing the lead apron between procedures).	**57% prevalence of back pain and 46% prevalence of neck pain.** **The majority of respondents (61%) wore a one-piece lead apron while performing ERCP.**
**Orme et al., 2015** [17] USA	Case-control study To determine whether the prevalence of work-related musculoskeletal pain and other medical conditions is higher among physicians and allied staff who work in interventional laboratories (require wearing lead aprons and exposure to radiation), compared with employees who do not.	Employees of Mayo Clinic: - 1543 completed the survey (response rate of 57%), - 1042 involved with procedures utilising radiation. - Mean age 43 ± 11.3 y - 33% M	Web-based Survey	**Clinical employees with occupational exposure to procedures involving radiation requiring lead apron use reported experiencing work-related pain more often than the control group (54.7% vs. 44.7%; *p* < 0.001) and after adjustment for age, sex, body mass index, pre-existing musculoskeletal conditions, years in profession, and job description (odds ratio: 1.67; 95% confidence interval: 1.32 to 2.11; *p* < 0.001).**
**Ross et al., 1997** [18] USA	Cross-sectional study To investigate the relationship between lead radiation shielding aprons and frequency of back pain, neck pain, and sciatica.	385 interventional cardiologists - Mean age 46 ± 8 y - 95.3% M 131 orthopedists - Mean age 49.9 ± 10.9 y - 93.9% Male 198 rheumatologists Mean age 45.4 ± 7.4 y 71.1% Male	Survey conducted by self-administered 16-item questionnaire, including: - age and gender - occurrence of back pain or sciatica before specialty training, years of practice, average number of procedures performed requiring X-ray per week - number of hours per day wearing lead aprons in an average week, use of 1- vs. 2-piece aprons - missed work days secondary to back or leg pain - number of days missed in the prior 12 months - use of conservative therapy including bed rest and/or support devices, analgesics and/or muscle relaxants for back or leg pain, surgical procedure for herniated disc, the type of procedure, and the intervertebral disc level.	6.5% prevalence of cervical disk herniation in the cardiology group, compared to 0.3% in the orthopedic surgeons and 0% in the rheumatologists (*p* < 0.001). Multiple level disc diseases: 3.4% prevalence in the cardiology group, compared to 0% in the orthopedic surgeons and 0% in the rheumatologists; (*p* < 0.03). The percentage of cardiologists, orthopedic surgeons, and rheumatologists who reported the use of aprons was 99.7%, 82.4%, and 5.0%, respectively (*p* < 0.001 cardiologists vs. the other two groups). One-piece aprons were worn by most physicians doing procedures in all groups, with 2-piece aprons worn by 22.9% of cardiologists, and none in orthopedic surgeons. The average number of reported radiologic procedures per week was 12.1 for cardiologists, 2.9 for orthopedic surgeons, and 0.6 for rheumatologists. The average reported hours per day aprons were worn were 8.4 by cardiologists, 2.0 for orthopedic surgeons, and 0.2 for rheumatologists (*p* < 0.0001). **The authors observed that cardiologists who wore aprons for considerably longer periods of time had a substantially greater frequency of skeletal complaints and more missed days from work due to pain, compared with the control groups.**
**Rothmore et al., 2002** [19] Australia	Cross-sectional study To compare body part discomfort ratings, fatigue, and ease of movement among radiographers while wearing two-piece lead suits, one-piece suits, and one-piece suits with waist belts.	Five angiographers 3 F; 2 M full-time employed	Workers enrolled used three different lead apron types (two-piece suits, one-piece suits, one-piece suits with waist belts) on two occasions. They were asked to indicate their level of discomfort by compilating a visual analogue scale (VAS) on their perceived levels of discomfort and fatigue at the beginning (T1) and end (T2) of patient procedural lists.	**While wearing a one-piece apron, significant differences were found in levels of discomfort between T1 and T2 in the neck/shoulders (*p* < 0.05) and lower back (*p* < 0.05).** **Wilcoxon tests showed that subjects experienced significantly greater levels of discomfort at T2 and a significant increase in fatigue levels between T1 and T2 for subjects while wearing a one-piece lead apron (*p* < 0.05).** **A Friedman’s test (*p* = 0.07), indicated a trend towards decreased ease of movement for subjects while wearing a one-piece lead apron.**
**Tetteh et al., 2020** [20] USA	Cross-sectional study To determine the effects of radiation personal protective equipment (rPPE) on the development of fatigue of the erector spinae and trapezius muscles while performing a simulated surgical procedure.		Surface EMG for recording muscle activity from trapezius and erector spinae muscles.	**The results from the statistical analysis showed that the rPPE significantly accelerated fatigue development in several of the muscles sampled.**

Ref. no = Reference number; MSDs = Musculoskeletal disorders; HCWs = healthcare workers; F = female; M = male; ERCP= Endoscopic retrograde Cholangiopancreatography.; OR_M-H_= Odds ratio Mantel-Heanszel; sEMG = surface electromyography.

## Abbreviations

The following abbreviations are used in this manuscript:

HCWs	Healthcare workers
MSDs	Musculoskeletal disorders
ERCP	Endoscopic retrograde cholangiopancreatography
LBP	Low back pain
BP	Back pain
NP	Neck pain
